# Furobenzotropolones A, B and 3-Hydroxyepicoccone B with Antioxidative Activity from Mangrove Endophytic Fungus *Epicoccum nigrum* MLY-3

**DOI:** 10.3390/md19070395

**Published:** 2021-07-14

**Authors:** Ge Zou, Qi Tan, Yan Chen, Wencong Yang, Zhenming Zang, Hongming Jiang, Shenyu Chen, Bo Wang, Zhigang She

**Affiliations:** 1 School of Chemistry, Sun Yat-Sen University, Guangzhou 510006, China; zoug5@mail2.sysu.edu.cn (G.Z.); tanq27@mail2.sysu.edu.cn (Q.T.); chenyan27@mail2.sysu.edu.cn (Y.C.); yangwc6@mail2.sysu.edu.cn (W.Y.); zangzhm@mail2.sysu.edu.cn (Z.Z.); jianghm7@mail2.sysu.edu.cn (H.J.); chensy87@mail2.sysu.edu.cn (S.C.); 2 National R & D Center for Edible Fungus Processing Technology, Henan University, Kaifeng 475004, China

**Keywords:** mangrove endophytic fungus, *Epicoccum nigrum*, furobenzotropolone, DPPH· scavenging activity, ABTS· scavenging activity

## Abstract

Three new metabolites, furobenzotropolones A, B (**1**–**2**) with unusual benzene and dihydrofuran moieties and 3-hydroxyepicoccone B (**3**), together with seven known compounds (**4**–**10**) were obtained from the endophytic fungus *Epicoccum nigrum* MLY-3 isolated from the fresh leaf of mangrove plant *Bruguiear gymnorrhiza* collected from Zhuhai. Their structures were assigned by the analysis of UV, IR, NMR, and mass spectroscopic data. Compound **1** was further confirmed by single-crystal X-ray diffraction experiment using Cu K*α* radiation. In antioxidant activities in vitro, compounds **2**, **3**, **5**, and **8** showed promising DPPH· scavenging activity with IC_50_ values ranging from 14.7 to 29.3 µM. Compounds **2**, **3**, **5**, **7**, and **8** exhibited promising potent activity in scavenging ABTS· with IC_50_ values in the range of 18–29.2 µM, which was stronger than that of the positive control ascorbic acid (IC_50_ = 33.6 ± 0.8 µM).

## 1. Introduction

Troponoids are characterized by a unique cyclohepta-2,4,6-trienone moiety, which is a seven-membered non-benzenoid aromatic ring, and troponoids are based on two simple seven-membered ring structures, tropone and tropolone [1,2]. Tropolones are usually substituted at C-2 with a hydroxyl group, incorporating various side chains on the seven-membered aromatic ring, such as acetyl, hydroxyl, isopropyl, alkaloids, flavonoids, and terpenoids [3]. Tropolones are known to be produced by fungi, bacteria, and plants [4,5,6], and these compounds have multiple bioactivities including antimicrobial, antiviral, anti-inflammatory, antitumor, and polyphenol oxidase inhibition activities [7,8,9,10,11].

**Figure 1:**
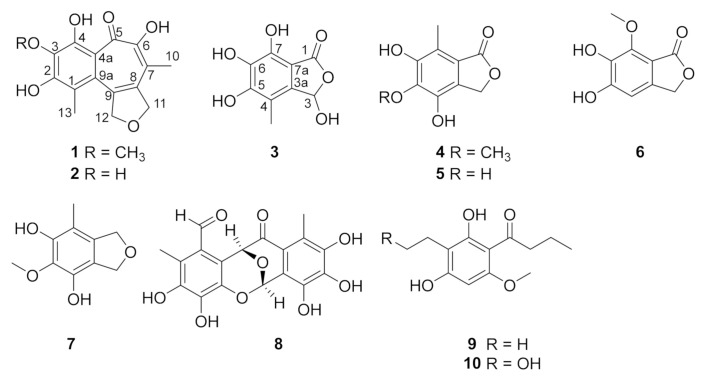
The structures of **1**–**10**.

In recent years, a series of novel bioactive compounds was dedicated from mangrove endophytic fungi in our group [12,13,14,15,16]. Recently, a chemical investigation focusing on the mangrove endophytic fungus *Epicoccum nigrum* MLY-3, which was insulated from the fresh leaf of mangrove plant *Bruguiear gymnorrhiza*, led to the isolation and characterization of three new metabolites (Figure 1), furobenzotropolones A, B (**1**–**2**) and 3-hydroxyepicoccone B (**3**), as well as seven previously reported compounds, 4,6-dihydroxy-5-methoxy-7-methylphthalide (**4**) [17], 4,5,6-trihydroxy-7-methyl-3*H*-isobenzofuran-1-one (**5**) [18], sparalide C (**6**) [19], 4,6-dihydroxy-5-methoxy-7-methyl-1,3-dihydroisobenzofuran (**7**) [20], epicoccolide A (**8**) [21], deoxyphomalone (**9**) [22], and phomalone (**10**) [22]. Their structures were established by extensive spectroscopic data (see Supplementary Materials) and comparison with the literature. In the bioactivity assays, all of the isolated compounds were evaluated for their DPPH· radical and ABTS· radical scavenging activities. Herein, the isolation, structure elucidation, DPPH· radical, and ABTS· radical scavenging activity of these compounds are reported.

## 2. Results

### 2.1. Structure Elucidation

Compound **1** was obtained as yellow amorphous powder. Its molecular formula was assigned as C_16_H_16_O_6_ according to HRESIMS analysis at *m*/*z* 303.08759 [M−H]^−^ (calcd. for C_16_H_15_O_6_, 303.08741), which was thus determined to possess nine degrees of unsaturation. In the ^1^H NMR spectrum, the signal for three hydroxyl protons at *δ*_H_ 14.71 (s, 4-OH), 10.22 (brs, 2-OH), and 9.46 (s, 6-OH), two methylene protons at *δ*_H_ 4.97 (s, H-11) and 5.10 (s, H-12), one methoxy proton at *δ*_H_ 3.80 (s, 3-OMe), and two methyls protons at *δ*_H_ 2.07 (s, H-10) and 2.17 (s, H-13) were observed (Table 1). In addition, according to the DEPT 135 and HSQC (Heteronuclear Single Quantum Correlation) data, the ^13^C NMR data showed the presence of 16 carbon signals, including one carbonyl (*δ*_C_ 184.3), ten sp^2^-hybridized quaternary carbons, two oxygenated sp^3^-hybridized carbons (*δ*_C_ 75.6 and *δ*_C_ 78.0), and two methyls (*δ*_C_ 14.7 and 15.1).

**Figure 2:**
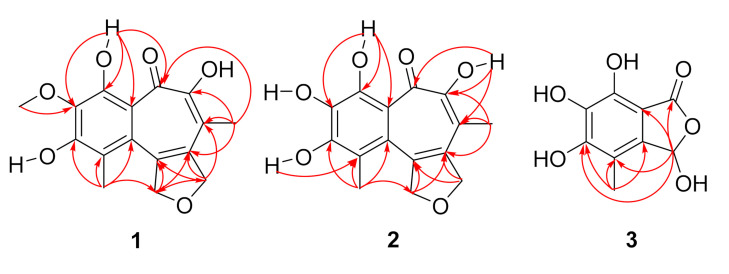
Key HMBC (red arrows) correlations of **1**–**3.**

**Figure 3:**
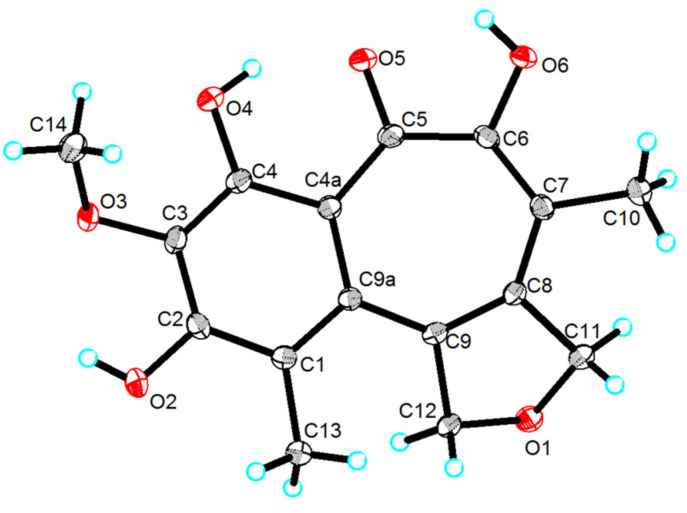
Single-crystal X-ray structures of **1.**

The HMBC (Heteronuclear Multiple Bond Correlation) correlations from 4-OH to C-3, C-4 and C-4a, from 3-OMe to C-3, and from H-13 to C-1, C-2, and C-9a constructed a 3-methoxy-1-methylbenzene ring fragment (Figure 2). Additionally, the HMBC correlations from H-10 to C-5, C-6, C-7, and C-8, from the H-11 to C-7, C-8, and C-9, from H-12 to C-8, C-9, from H-13 to C-9a, C-12, and from 4-OH to C-4a, C-5 assembled a tropolone ring, which connected with the benzene ring at C-4a and C-9a. Furthermore, HMBC correlations of H-11 to C-12, and H-12 to C-11 and unsaturation information indicated that **1** had a dihydrofuran ring, which was assigned to connect with the benzotropolone ring at C-8 and C-9. Thus, with the assistance of single-crystal X-ray (Figure 3), the structure of **1** was deduced and named furobenzotropolone A.

Compound **2** was obtained as a yellow amorphous powder. The molecular formula C_15_H_14_O_6_ was established on the basis of HRESIMS data at *m*/*z* 289.07185 [M−H]^−^ (calcd. for C_15_H_13_O_6_, 289.07176), which was thus determined to possess nine degrees of unsaturation. The ^1^H and ^13^C NMR spectroscopic data were listed in Table 1, which suggested that structure of **2** was similar with that of **1**, except that the methoxy group was substituted with the hydroxyl group at C-3. Combined with HMQC and HMBC (Figure 2), the structure of compound **2** was clearly confirmed, which was named furobenzotropolone B.

Compounds **1** and **2** possess unusual benzene and dihydrofuran moieties. According to the literature survey, similar structures included benzotropolones, such as purpurogallin, fomentariol, goupiolone A, aurantricholone, Crocipodin [23,24,25,26,27]; and furotropolones: nemanolone D, nemanolone E, and viticolins C, for instance [28,29], and there was no tropolone containing both benzene and dihydrofuran moieties reported previously.

Compound **3** was isolated as white amorphous powder. Its molecular formula was determined as C_9_H_8_O_6_ (six degrees of unsaturation) in terms of HREIMS analysis at *m*/*z* 211.02518 [M−H]^−^ (calcd. for C_9_H_7_O_6_, 211.02481). Analysis of the ^1^H and ^13^C NMR spectroscopic data of **3** (Table 2) revealed most similarities to those in the literature, rather than the methoxyl signal substituted at C-3 by a hydroxyl group [30]. Combined with HMQC and HMBC (Figure 2), compound **3** was determined as 3-hydroxyepicoccone B. The specific optical rotation value ${\left[\alpha \right]}_{D}^{20}$+ 1.2) of **3** indicates it to be a scalemic mixture.

### 2.2. Antioxidant Activities In Vitro

Compounds **1**–**10** were tested for their antioxidant activities in vitro. As seen in Table 3, the results indicated that compounds **2**, **3**, **5**, and **8** showed promising DPPH· scavenging activity with IC_50_ values of 26.5, 29.3, 16.5, and 14.7 µM, respectively, of which compounds **5** and **8** were better than the positive control ascorbic acid (20.1 µM). Compounds **1**, **4**, and **7** also exhibited weak DPPH· scavenging activity with respective IC_50_ values of 57.6, 85.2, and 53.1 µM. Beyond that, compounds **2**, **3**, **5**, **7**, and **8** possessed more potent ABTS· scavenging activity than the positive control with IC_50_ values of 29.2, 23.7, 23.3, 24.0, and 18.8 µM. Compounds **1**, **4**, and **6** also showed weak ABTS· scavenging activity with IC_50_ values of 46.4, 43.1, and 93.5 µM, respectively. Through the analysis of the structure–activity relationship, we found that antioxidant activity increased with the increase of phenolic hydroxyl groups. If the phenolic hydroxyl group is replaced with a methoxy group, the antioxidant activity will significantly decrease.

## 3. Experimental Section

### 3.1. General Experimental Procedures

Optical rotations were tested on an MCP300 (Anton Paar, Shanghai, China). UV data were recorded using a Shimadzu UV-2600 spectrophotometer (Shimadzu, Kyoto, Japan). IR spectra were recorded on IR Affinity-1 spectrometer (Shimadzu, Kyoto, Japan). The NMR spectra were recorded on a Bruker Avance spectrometer (Bruker, Beijing, China) (compounds **2**: 500 MHz for ^1^H and 125 MHz for ^13^C, respectively; compounds **1** and **3**: 400 MHz for ^1^H and 100 MHz for ^13^C). HRESIMS data were conducted on an Ion Mobility-Q-TOF High-Resolution LC-MS (Synapt G2-Si, Waters, Milford, MA, USA). Single-crystal data were measured on an Agilent Gemini Ultra diffractometer (Cu K*α* radiation, Agilent, Santa Clara, CA, USA). Column chromatography (CC) was performed on silica gel (200–300 mesh, Marine Chemical Factory, Qingdao, China) and Sephadex LH-20 (Amersham Pharmacia, Piscataway, NJ, USA).

### 3.2. Fungal Material

The fungal strain MLY-3 used in this study was isolated from fresh leaf of *Bruguiear gymnorrhiza*, which was collected from the Dongzhaigang Mangrove National Nature Reserve in Zhuhai, China, in April 2018. The strain was identified as *Epicoccum nigrum* (compared to no. MW081246.1) upon the analysis of ITS sequence data of the rDNA gene. The ITS sequence data obtained from the fungal strain have been submitted to GenBank with accession no. MZ407636. A voucher strain was deposited in our laboratory.

### 3.3. Fermentation, Extraction, and Isolation

The fungus *Epicoccum nigrum* MLY-3 was fermented on solid cultured medium (sixty 1000 mL Erlenmeyer flasks, each containing 50 g of rice and 50 mL 3‰ of saline water) for 30 days at 25 °C. The cultures were extracted three times with MeOH to yield 10.5 g of residue. Then, the crude extract was eluted by using gradient elution with petroleum ether/EtOAc from 9:1 to 0:10 (*v/v*) on silica gel CC to get six fractions (Fr.1–Fr.6). Fr.3 (630 mg) was separated to Sephadex LH-20 column (110 × 6 cm) chromatography, eluted with CH_2_Cl_2_-MeOH (1:1), to obtain five subfractions (Fr.3.1–Fr.3.2). Fr.3.2 (110 mg) was further purified by silica gel (30 × 3 cm column) (gradient of petroleum ether and ethyl acetate from 50:1 to 9:1 to give **9** (12.7 mg). Fr.4 (420 mg) was applied to silica gel CC by petroleum ether and ethyl acetate from 9:1 to 8:2 to obtain Fr.4.1–Fr.4.3. Fr.4.1 (95 mg) was purified by Sephadex LH-20 CC and eluted with MeOH to obtain compounds **1** (7 mg) and **4** (26.4 mg); Fr.4.2 (79 mg) was further purified by Sephadex LH-20 CC using MeOH to obtain compounds **6** (6.5 mg) and **10** (20.5 mg); Fr.4.3 (108.0 mg) was purified by Sephadex LH-20 CC using CH_2_Cl_2_/MeOH (1:1) to yield compound **7** (25.5 mg). Fr.5 (267 mg) was applied to silica gel CC by petroleum ether and ethyl acetate from 9:1 to 7:3 to obtain Fr.4.1–Fr.4.3. Fr.4.1 (86 mg), Fr.4.2 (32 mg), and Fr.4.3 (76 mg) were purified by Sephadex LH-20 CC using CH_2_Cl_2_/MeOH (1:1) to yield compounds **3** (7.9 mg), **2** (1.0 mg), and **8** (19.9 mg). Fr.6 (68 mg) was applied to silica gel CC by CH_2_Cl_2_/MeOH (20:1) to obtain compound **5** (6.7 mg).

Furobenzotropolone A (**1**): yellow, amorphous powder; UV (MeOH) *λ*_max_ (log *ε*): 296 (0.89), 275 (0.74), 219 (1.37), 204 (1.40) nm; IR (KBr) *υ*_max_ 3385, 2922, 1716, 1616, 1506, 1456, 1306, 1184, 1038, 717 cm^−1^; HRESIMS *m*/*z* 303.08759 [M−H]^−^ (calcd. for C_16_H_15_O_6_, 303.08741), ^1^H NMR and ^13^C NMR data: see Table 1.

Furobenzotropolone B (**2**): yellow, amorphous powder; UV (MeOH) *λ*_max_ (log *ε*): 309 (0.76), 278 (0.67), 218 (1.25), 205 (1.27) nm; IR (KBr) *υ*_max_ 3366, 2924, 1717, 1635, 1506, 1456, 1020, 972 cm^−1^; HRESIMS *m*/*z* 289.07185 [M−H]^−^ (calcd. for C_15_H_13_O_6_, 289.07176), ^1^H NMR and ^13^C NMR data: see Table 1.

3-hydroxyepicoccone B (**3**): White powder; ${\left[\alpha \right]}_{D}^{25}$+ 1.2 (c 0.04 MeOH); UV(MeOH) *λ*_max_ (log ε): 270 (0.71), 221 (1.90) nm; IR (KBr) *υ*_max_ 3313, 2931, 1716, 1636, 1519, 1489, 1269, 1016, 912, 868 cm^−1^; HRESIMS *m*/*z* 211.02518 [M−H]^−^ (calcd for C_9_H_7_O_6_, 211.02481), ^1^H NMR and ^13^C NMR data: see Table 2.

### 3.4. X-Ray Crystallographic Data

Yellow crystal of compound **1** was obtained from MeOH-CH_2_Cl_2_ at room temperature by slow evaporation and measured on an Agilent Xcalibur Nova single crystal diffractometer with Cu K*α* radiation.

The crystallographic data for compound **1** have been deposited in the Cambridge Crystallographic Data Centre (CCDC number: 2091599).

Crystal data of **1**: C_16_H_16_O_6_, *Mr* = 304.29, monoclinic, a = 3.9051(6) Å, b = 8.3456(13) Å, c = 40.256(6) Å, *α* = 90°, *β* = 90.84°, *γ* = 90°, V = 1311.8(4) Å3; space group P2_1_/n, Z = 4, T = 150 K, Dc = 1.541 g/cm^3^, μ = 0.998 mm^–1^, and F(000) = 604.0. Crystal dimensions: 0.2 × 0.2 × 0.1 mm^3^. Independent reflections: 2618 (R_int_ = 0.0321). The final *R*_1_ values were 0.0417, w*R*_2_ = 0.1221 [I >= 2σ (*I*)]. The goodness of fit on *F*^2^ was 0.999.

### 3.5. Antioxidant Activity Analysis

#### 3.5.1. DPPH· (2, 2-diphenyl-1-picrylhydrazyl) Scavenging Activity

The DPPH· radical scavenging capacities of compounds **1**–**10** were determined utilizing the reported method [31]. The DPPH· radical scavenging test was performed in 96-well microplates. Samples (100 µL) with a final concentration range of 6.25–100 µM were added to 100 µL of 0.16 mM DPPH· in MeOH. An ascorbic acid positive control was prepared at the same concentrations as the test samples (Table 3). Absorbance was measured at λ = 517 nm after 30 min of incubation in the dark. The DPPH· radical scavenging activity was calculated using the formula:

DPPH· radical scavenging activity (%) = [(Abs _control_ − Abs _sample_)/Abs _control_] × 100.

#### 3.5.2. ABTS· (2,2′-azino-bis(3-ethylbenzthiazoline-6-sulfonic acid) Radical Cation Scavenging Activity

The ABTS· scavenging activity of compounds **1**–**10** was also resolved according to the ABTS· method (Beyotime Institute of Biotechnology, China) with a slight modification. In brief, ABTS· radical cation solution was prepared by mixing ABTS· solution with oxidant solution in equal quantities and allowing them to react in the dark at room temperature for 16 h before use. Then, the solution was diluted by mixing 1 mL working solution with 20 mL of 80% ethanol. A fresh ABTS· solution was prepared for each assay. Samples (100 µL) with a final concentration range of 12.5–100 µM were mixed with 100 µL of fresh ABTS· solution, and the mixture was left at room temperature for 6 min. Then, the absorbance was measured at 734 nm. Ascorbic acid was used as a reference compound. The ABTS· radical scavenging activity was calculated as follows:

ABTS· radical scavenging activity (%) = [(Abs _control_ − Abs _sample_)/Abs _control_] × 100.

## 4. Conclusions

In summary, three new metabolites, furobenzotropolones A, B (**1**–**2**), 3-hydroxyepicoccone B (**3**), and seven known compounds were isolated from the fungus *Epicoccum nigrum* MLY-3. Their structures were determined by the analysis of UV, IR, NMR, mass spectroscopic data, and single-crystal X-ray diffraction experiment. Compounds **1** and **2** possess unusual tropolone skeletons containing both benzene and dihydrofuran moieties. All of the compounds were tested for their antioxidant activities in vitro. Compounds **2**, **3**, **5**, and **8** showed promising DPPH· scavenging activity with IC_50_ values of 26.5, 29.3, 16.5, and 14.7 µM, respectively. Meanwhile, compounds **2**, **3**, **5**, **7**, and **8** possessed more potent capacity than positive control ascorbic acid in scavenging ABTS· with IC_50_ values of 29.2, 23.7, 23.3, 24.0 and 18.8 µM.

## Figures and Tables

**Table 1:** ^1^H and ^13^C NMR data for compounds **1** ^a^ and **2** ^b^.

	1		2	
No.	*δ*_C_, Type	*δ*_H_ Mult (*J* in Hz)	*δ*_C_, Type	*δ*_H_ Mult (*J* in Hz)
1	114.1, C		114.2, C	
2	154.5, C		150.6, C	
3	134.1, C		131.8, C	
4	154.6, C		149.0, C	
4a	117.2, C		116.6, C	
5	184.3, C		182.2, C	
6	151.4, C		152.1, C	
7	119.4, C		119.9, C	
8	135.9, C		135.1, C	
9	132.1, C		132.9, C	
9a	131.1, C		127.5, C	
10	14.7, CH_3_	2.07, s	14.9, CH_3_	2.10, s
11	75.6, CH_2_	4.97, s	75.5, CH_2_	4.97, s
12	78.0, CH_2_	5.10, s	78.2, CH_2_	5.13, s
13	15.1, CH_3_	2.17, s	15.1, CH_3_	2.19, s
2-OH		10.22, brs		10.08, s
3-OMe/-OH	59.8, CH_3_	3.80, s		9.50, s
4-OH		14.71, s		14.87, s
6-OH		9.46, s		9.35, s

^a^ Data were recorded in DMSO-*d*_6_ at 400 MHz for ^1^H NMR and 100 MHz for ^13^C NMR. ^b^ Data were recorded in DMSO-*d*_6_ at 500 MHz for ^1^H NMR and 125 MHz for ^13^C NMR.

**Table 2:** ^1^H and ^13^C NMR data for **3**^a^_._

	3	
No	*δ*_C_, Type	*δ*_H_ Mult (*J* in Hz)
1	171.6, C	
3	99.1, CH	6.45, s
3a	138.5, C	
4	114.0, C	
5	153.0, C	
6	143.8, C	
7	135.1, C	
7a	104.8, C	
7-CH_3_	10.6, CH_3_	2.17, s

^a^ Data were recorded in MeOH-*d*_4_ at 400 MHz for ^1^H NMR and 100 MHz for ^13^C NMR.

**Table 3:** DPPH· scavenging activity and ABTS· scavenging activity of compounds **1**–**10**.

Compound	DPPH· Scavenging Activity IC_50_ (μM)	ABTS· Scavenging Activity IC_50_ (μM)
1	57.6 ± 1.1	46.4 ± 1.6
2	26.5 ± 1.0	29.2 ± 0.9
3	29.3 ± 1.5	23.7 ± 0.6
4	85.2 ± 4.1	43.1 ±1.0
5	16.5 ± 0.9	23.3 ± 0.6
6	>100	93.5 ± 2.0
7	53.1 ± 0.7	24.0 ± 0.6
8	14.7 ± 0.4	18.8 ± 0.4
9	>100	>100
10	>100	>100
ascorbic acid ^a^	20.1 ± 0.32	33.6 ± 0.8

^a^ positive control.

## Supplementary Materials

The following are available online at https://www.mdpi.com/article/10.3390/md19070395/s1, Figure S1. HRESIMS spectrum of compound **1**, Figure S2. ^1^H NMR spectrum of compound **1** (400 MHz, DMSO-*d*_6_), Figure S3. ^13^C NMR spectrum of **1** (100 MHz, DMSO-*d*_6_), Figure S4. DEPT 135 and ^13^C NMR spectra of **1** (100 MHz, DMSO-*d*_6_), Figure S5. HSQC spectrum of compound **1** (DMSO-*d*_6_), Figure S6. HMBC spectrum of compound **1** (DMSO-*d*_6_), Figure S7. HRESIMS spectrum of compound **2**, Figure S8. ^1^H NMR spectrum of compound **2** (500 MHz, DMSO-*d*_6_), Figure S9. ^13^C NMR spectrum of **2** (125 MHz, DMSO-*d*_6_), Figure S10. DEPT 135 and ^13^C NMR spectra of **2** (125 MHz, DMSO-*d*_6_), Figure S11. HSQC spectrum of compound **2** (DMSO-*d*_6_), Figure S12. HMBC spectrum of compound **2** (DMSO-*d*_6_), Figure S13. HREIMS spectrum of compound **3**, Figure S14. ^1^H NMR spectrum of compound **3** (400 MHz, MeOH-*d*_4_), Figure S15. ^13^C NMR spectrum of **3** (100 MHz, MeOH-*d*_4_), Figure S16. HSQC spectrum of compound **3** (MeOH-*d*_4_), Figure S17. HMBC spectrum of compound **3** (MeOH-*d*_4_).
