# Supplementary material for: Furobenzotropolones A, B and 3-Hydroxyepicoccone B with Antioxidative Activity from Mangrove Endophytic Fungus Epicoccum nigrum MLY-3
Source: Mar Drugs. 2021 Jul 14;19(7):395. doi: 10.3390/md19070395 (PMC8304361; doi:10.3390/md19070395)
Supplement: Supplementary file 1 [file marinedrugs-19-00395-s001.zip › Supporting information.pdf]

# Supporting information

# Furobenzotropolones A, B and 3-hydroxyepicoccone B with Antioxidative Activity from Mangrove Endophytic Fungus *Epicoccum nigrum* MLY-3

Ge Zou [1], Qi Tan [1], Yan Chen [1,2], Wencong Yang [1], Zhenming Zang [1], Hongming Jiang [1], Shenyu Chen [1], Bo Wang [1,] * and Zhigang She [1,] *

1 School of Chemistry, Sun Yat-Sen University, Guangzhou 510006, China;

2 National R & D Center for Edible Fungus Processing Technology, Henan University, Kaifeng 475004, China;

* Correspondence: ceswb@mail.sysu.edu.cn (B. W.) cesshzhg@mail.sysu.edu.cn (Z. S.).

**Supporting information**


**Fig.S1.** HRESIMS spectrum of compound **1** 3

**Fig.S2.** $^{1}$H NMR spectrum of compound **1** (400 MHz, DMSO-$d_6$) 3

**Fig.S3.** $^{13}$C NMR spectrum of **1** (100 MHz, DMSO-$d_6$) 4

**Fig.S4.** DEPT 135 and $^{13}$C NMR spectra of **1** (100 MHz, DMSO-$d_6$) 4

**Fig.S5.** HSQC spectrum of compound **1** (DMSO-$d_6$) 5

**Fig.S6.** HMBC spectrum of compound **1** (DMSO-$d_6$) 5

**Fig.S7.** HRESIMS spectrum of compound **2** 6

**Fig.S8.** $^{1}$H NMR spectrum of compound **2** (500 MHz, DMSO-$d_6$) 6

**Fig.S9.** $^{13}$C NMR spectrum of **2** (125 MHz, DMSO-$d_6$) 7

**Fig.S10.** DEPT 135 and $^{13}$C NMR spectra of **2** (125 MHz, DMSO-$d_6$) 7

**Fig.S11** HSQC spectrum of compound **2** (DMSO-$d_6$) 8

**Fig.S12.** HMBC spectrum of compound **2** (DMSO-$d_6$) 8

**Fig.S13.** HREIMS spectrum of compound **3** 9

**Fig.S14.** $^{1}$H NMR spectrum of compound **3** (400 MHz, MeOH-$d_4$) 9

**Fig.S15.** $^{13}$C NMR spectrum of **3** (100 MHz, MeOH-$d_4$) 10

**Fig.S16.** HSQC spectrum of compound **3** (MeOH-$d_4$) 10

**Fig.S17.** HMBC spectrum of compound **3** (MeOH-$d_4$) 11

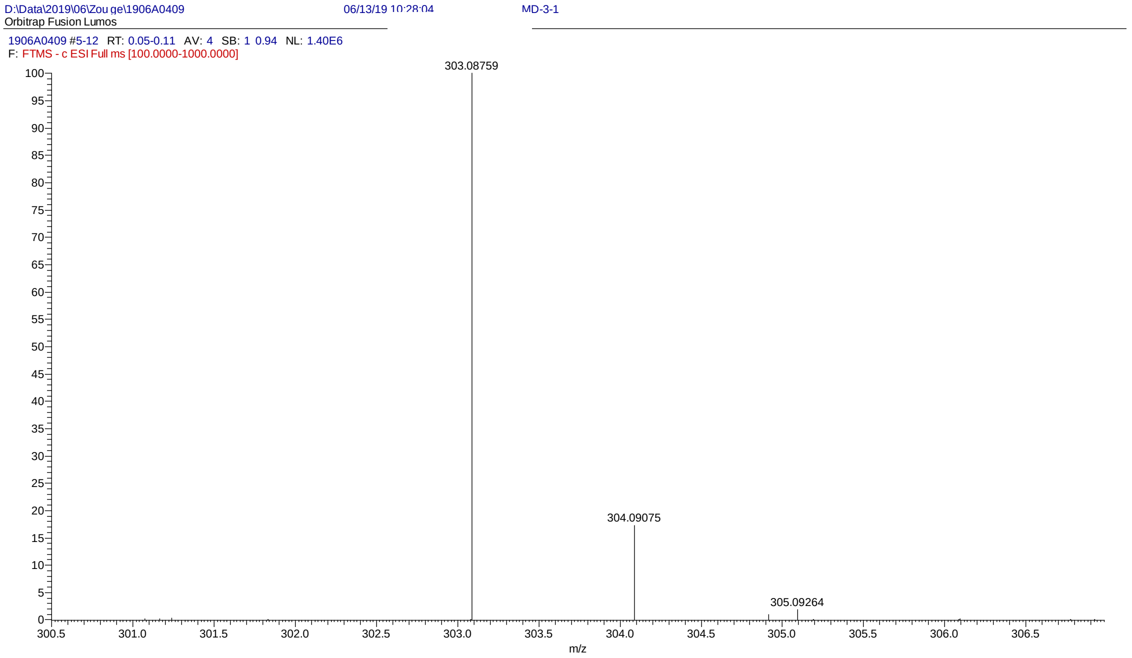


SPECTRUM - simulation :

| *m/z* | Theo. Mass | Delta (ppm) | RDB equiv. | Composition |
|---|---|---|---|---|
| 303.08759 | 303.08741 | 0.59 | 9.5 | $C_{16}$ $H_{15}$ $O_6$ |

**Fig.S1.** HRESIMS spectrum of compound **1**

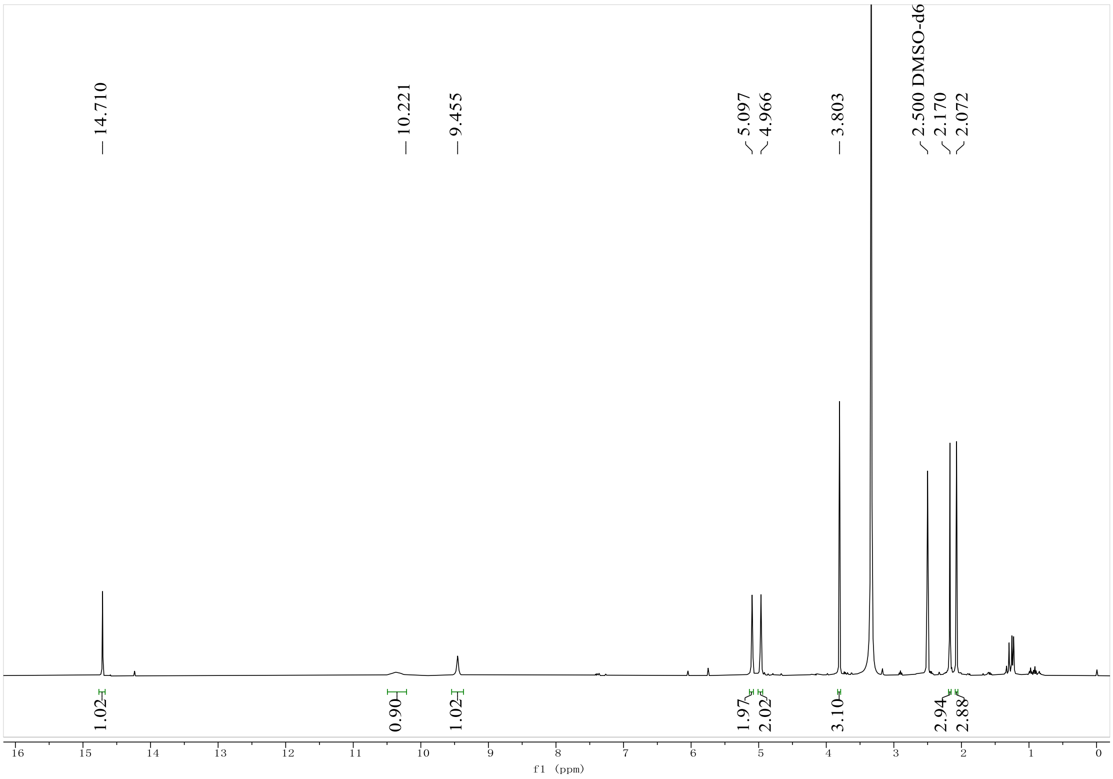


**Fig.S2.** $^{1}$H NMR spectrum of compound **1** (400 MHz, DMSO-$d_6$)

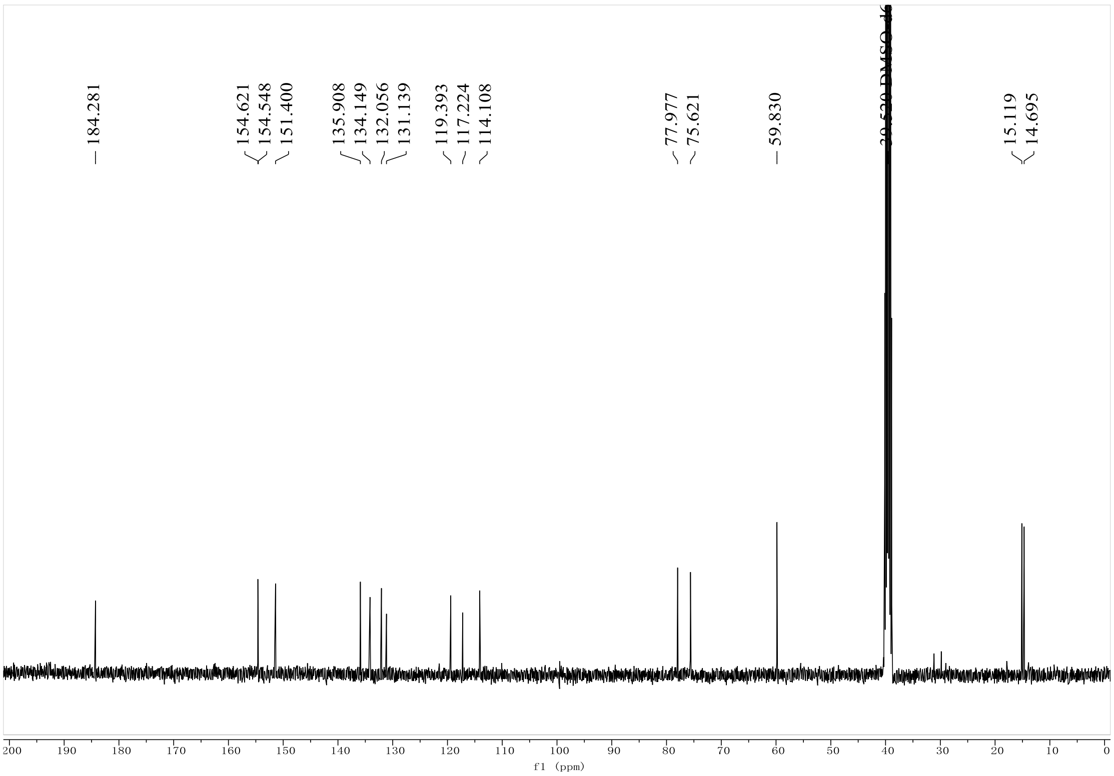


**Fig.S3.** $^{13}C$ NMR spectrum of compound **1** (100 MHz, DMSO-$d_6$)

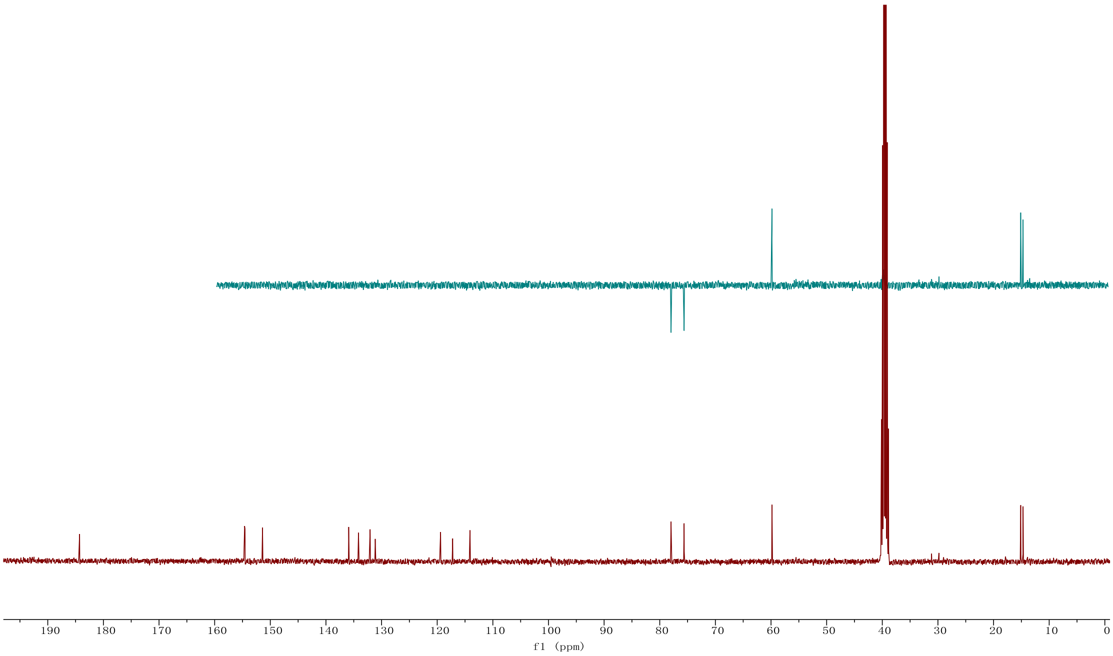


**Fig.S4.** DEPT 135 and $^{13}C$ NMR spectra of compound **1** (100 MHz, DMSO-$d_6$)

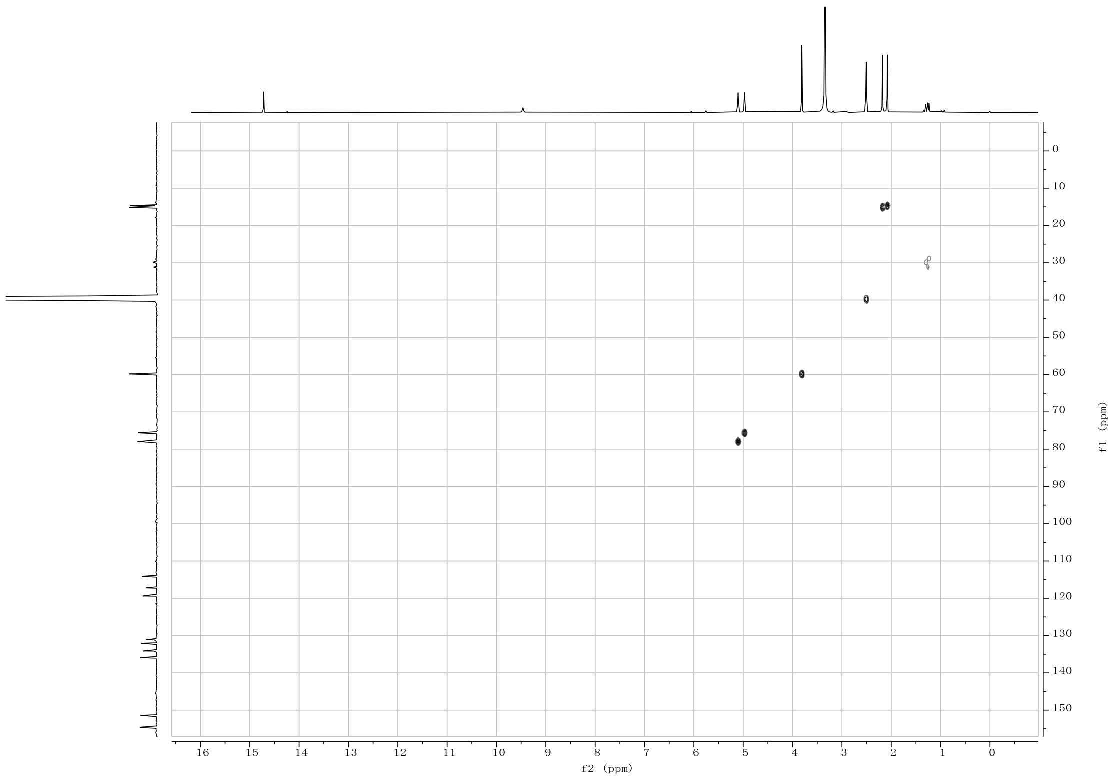


**Fig.S5.** HSQC spectrum of compound **1** (DMSO-$d_6$)

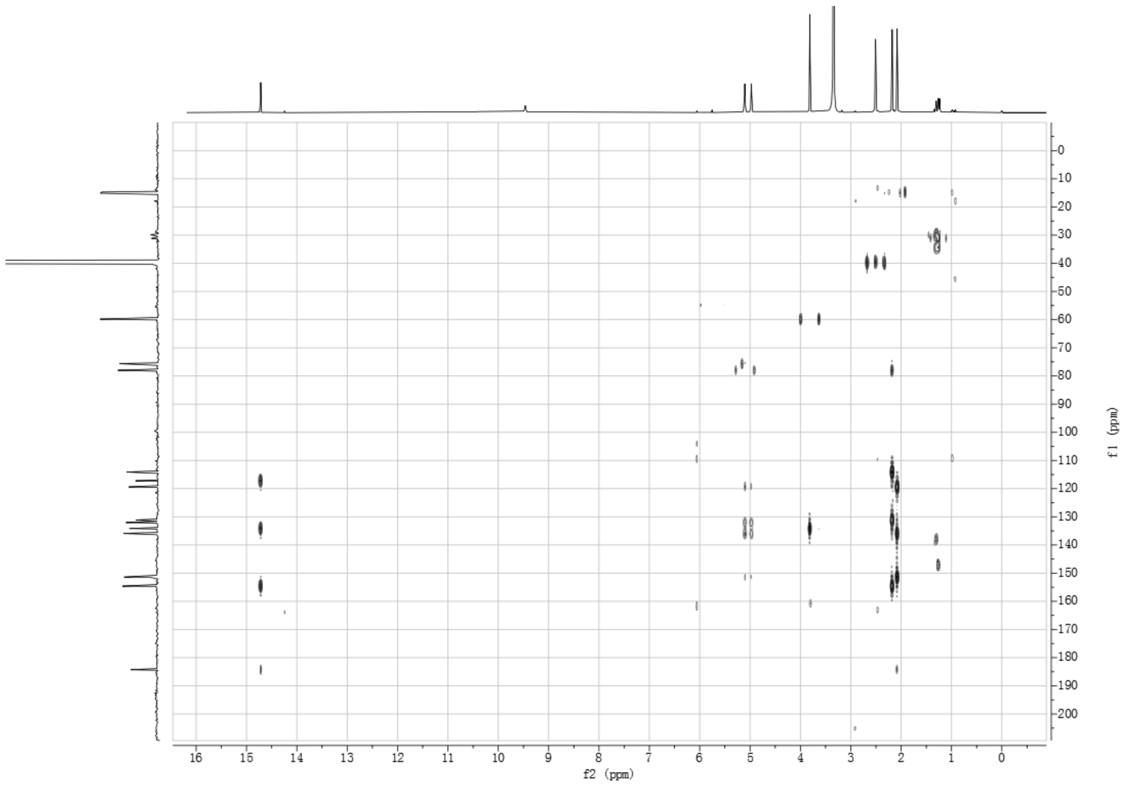


**Fig.S6.** HMBC spectrum of compound **1** (DMSO-$d_6$)

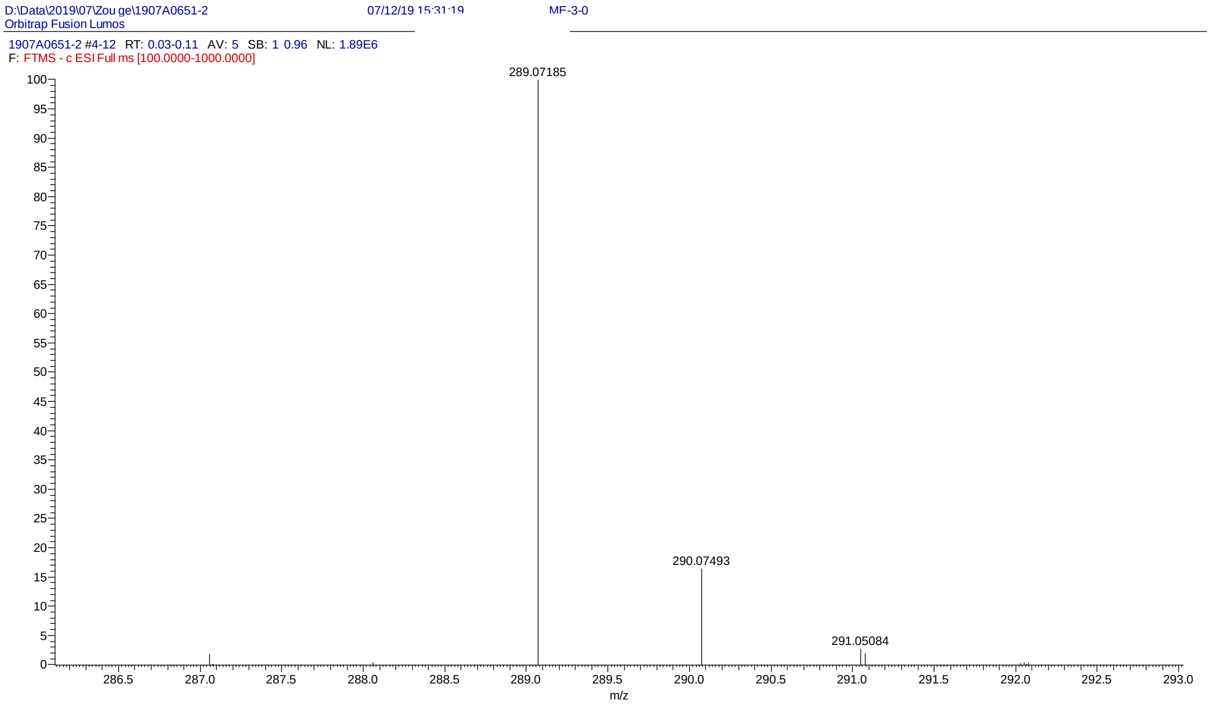


SPECTRUM - simulation :

| *m/z* | Theo. Mass | Delta (ppm) | RDB equiv. | Composition |
|---|---|---|---|---|
| 289.07185 | 289.07176 | 0.31 | 9.5 | $C_{15}H_{13}O_6$ |

**Fig.S7.** HRESIMS spectrum of compound **2**

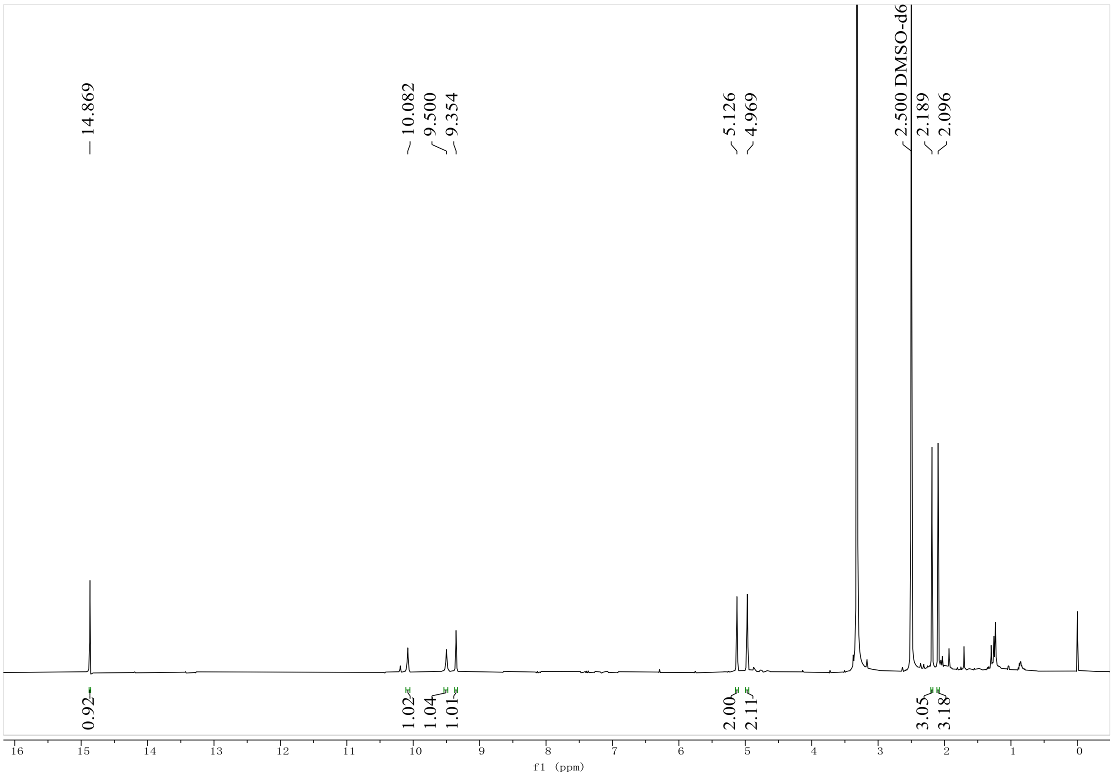


**Fig.S8.** $^{1}$H NMR spectrum of compound **2** (500 MHz, DMSO-$d_6$)

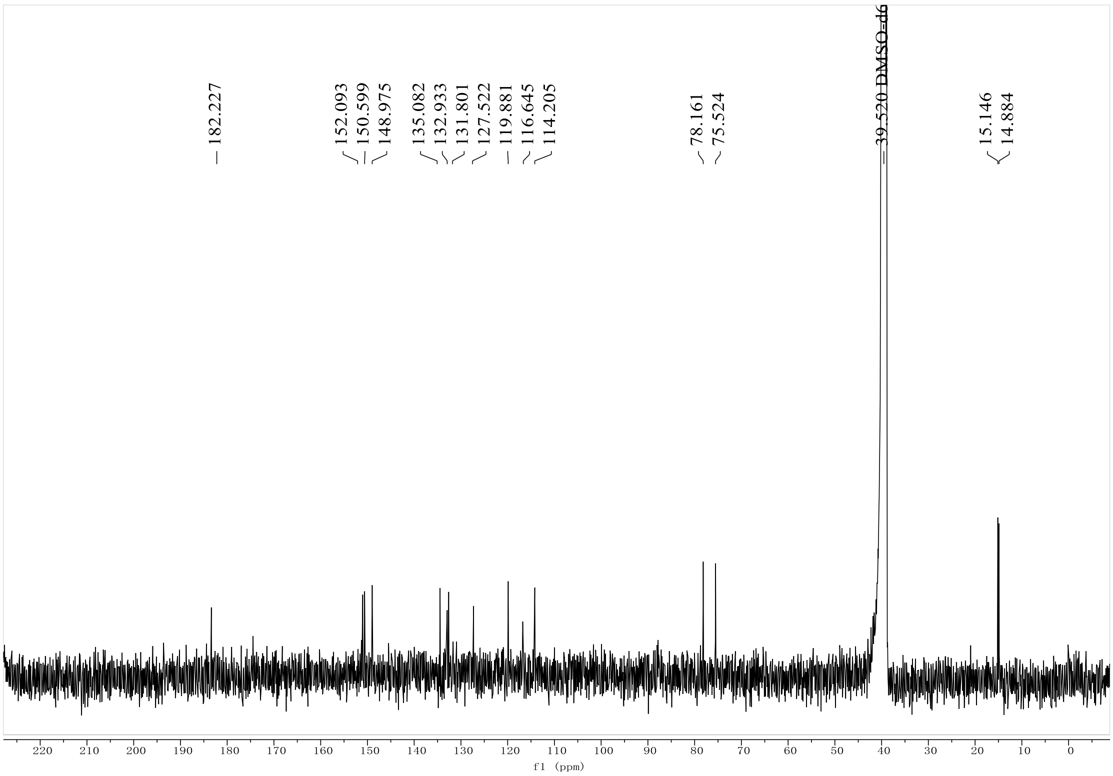


**Fig.S9.** $^{13}$C NMR spectrum of compound **2** (125 MHz, DMSO-$d_6$)

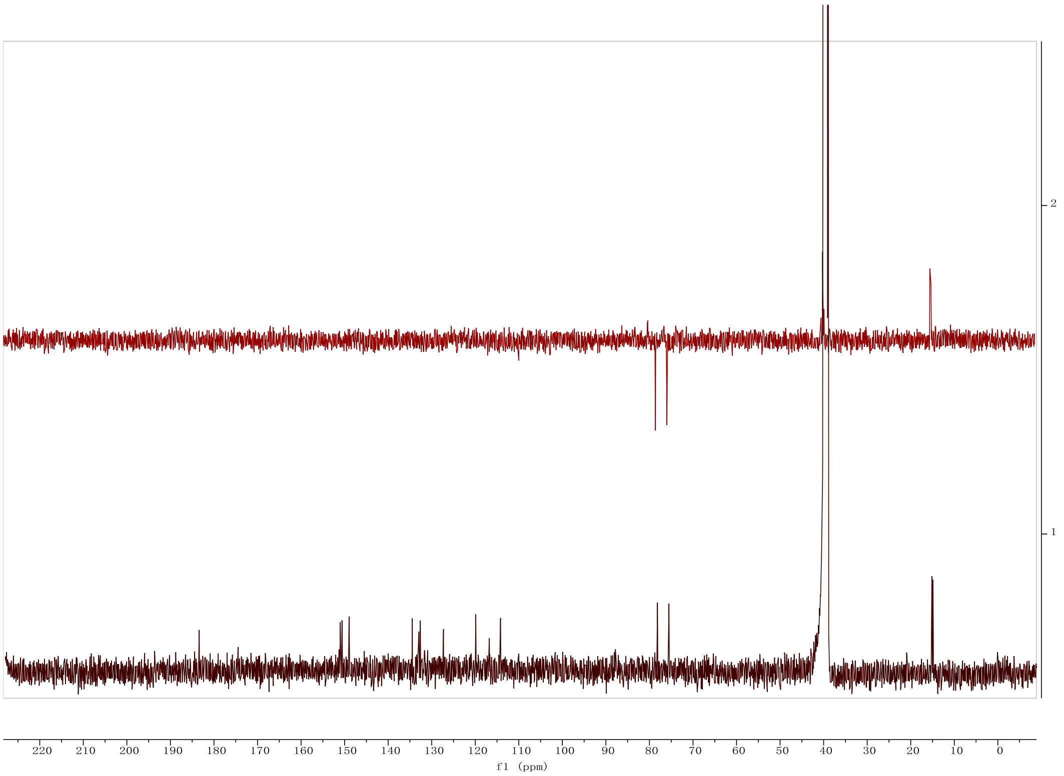


**Fig.S10.** DEPT 135 and $^{13}$C NMR spectra of compound **2** (125 MHz, DMSO-$d_6$)

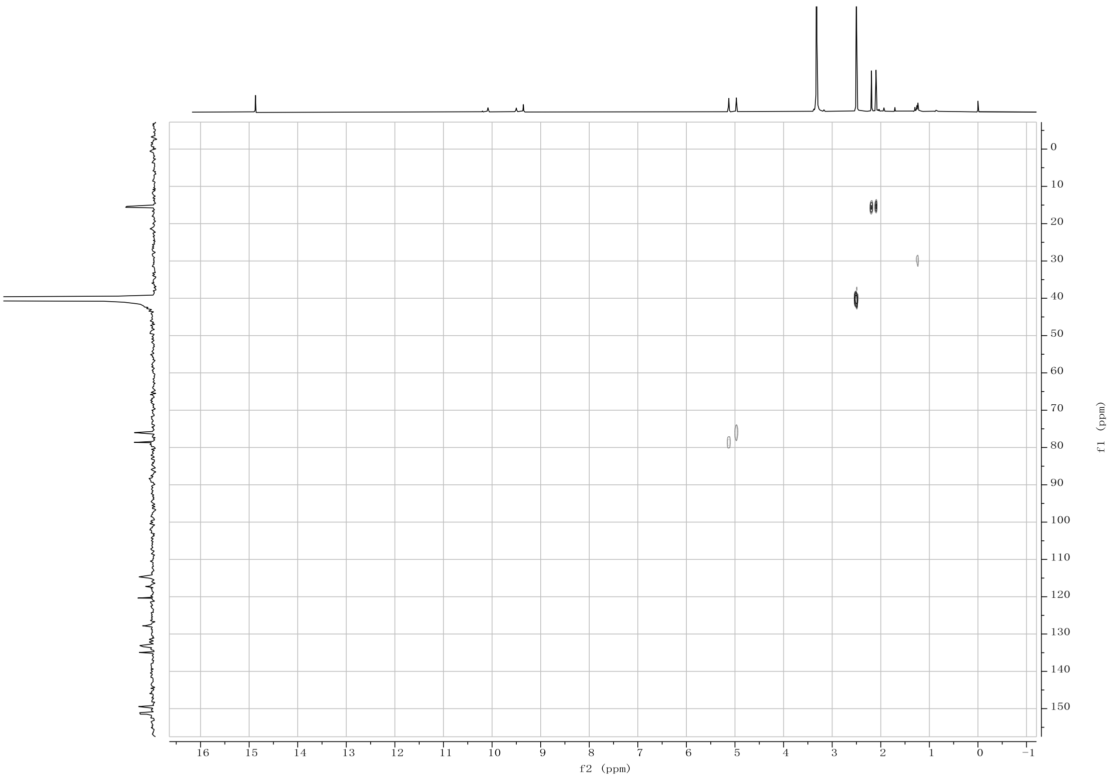


**Fig.S11.** HSQC spectrum of compound **2** (DMSO-$d_6$)

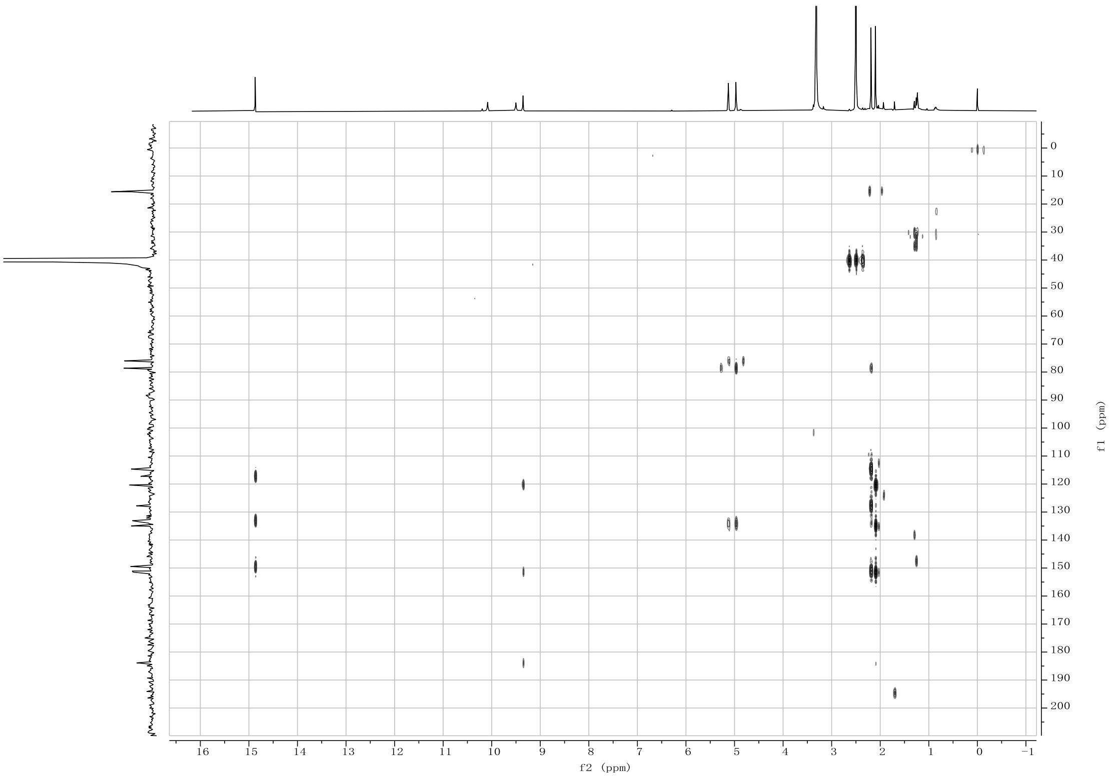


**Fig.S12.** HMBC spectrum of compound **2** (DMSO-$d_6$)

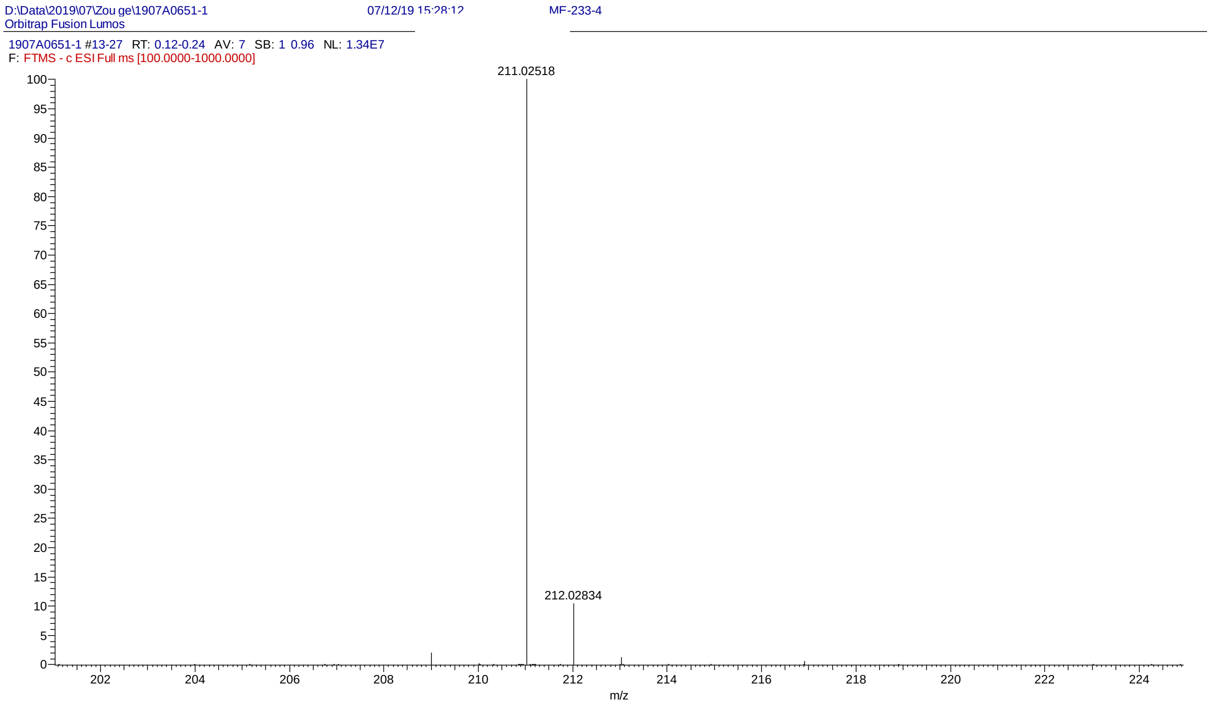


SPECTRUM - simulation :

| *m/z* | Theo. Mass | Delta (ppm) | RDB equiv. | Composition |
|---|---|---|---|---|
| 211.02518 | 211.02481 | 1.75 | 6.5 | $C_9H_7O_6$ |

**Fig.S13.** HRESIMS spectrum of compound **3**

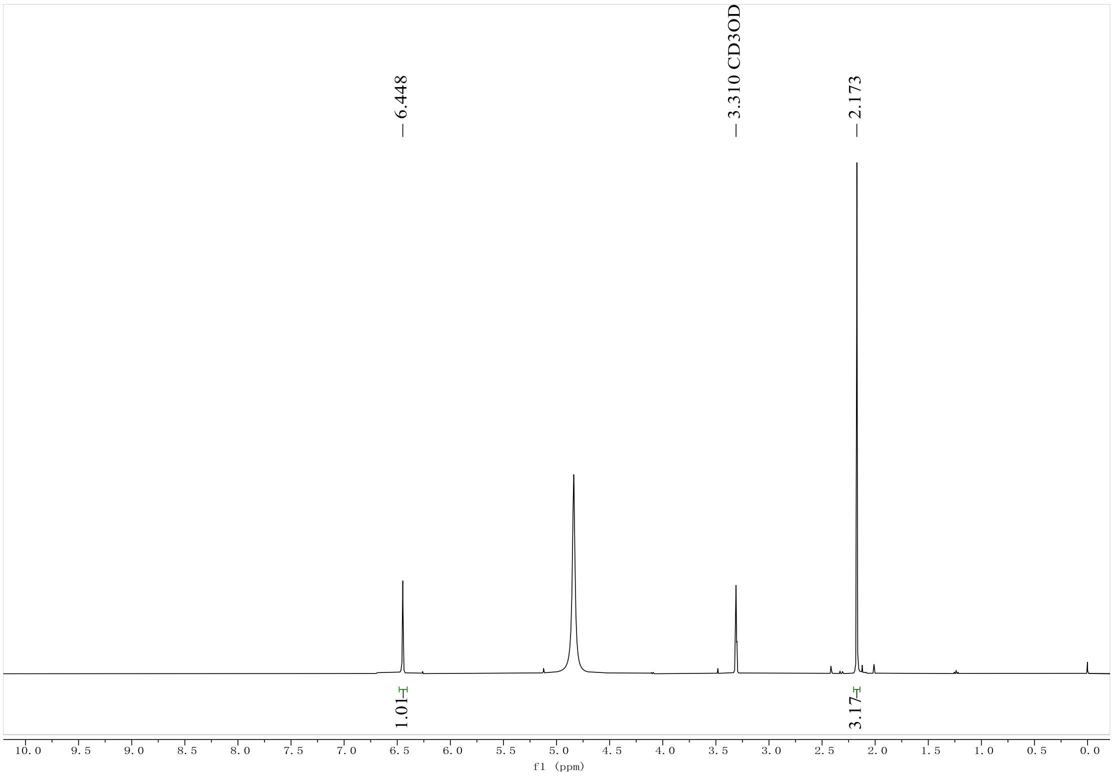


**Fig.S14.** $^{1}$H NMR spectrum of compound **3** (400 MHz, MeOH-$d_4$)

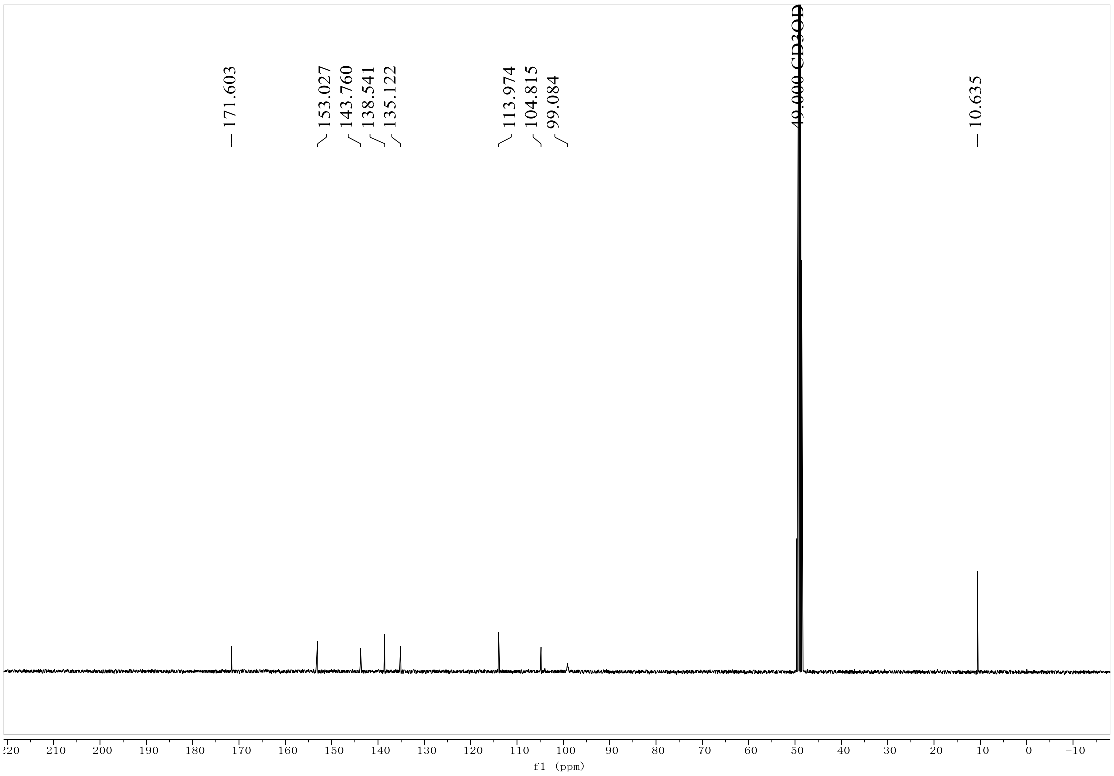


**Fig.S15.** $^{13}$C NMR spectrum of compound **3** (100 MHz, MeOH-$d_4$)

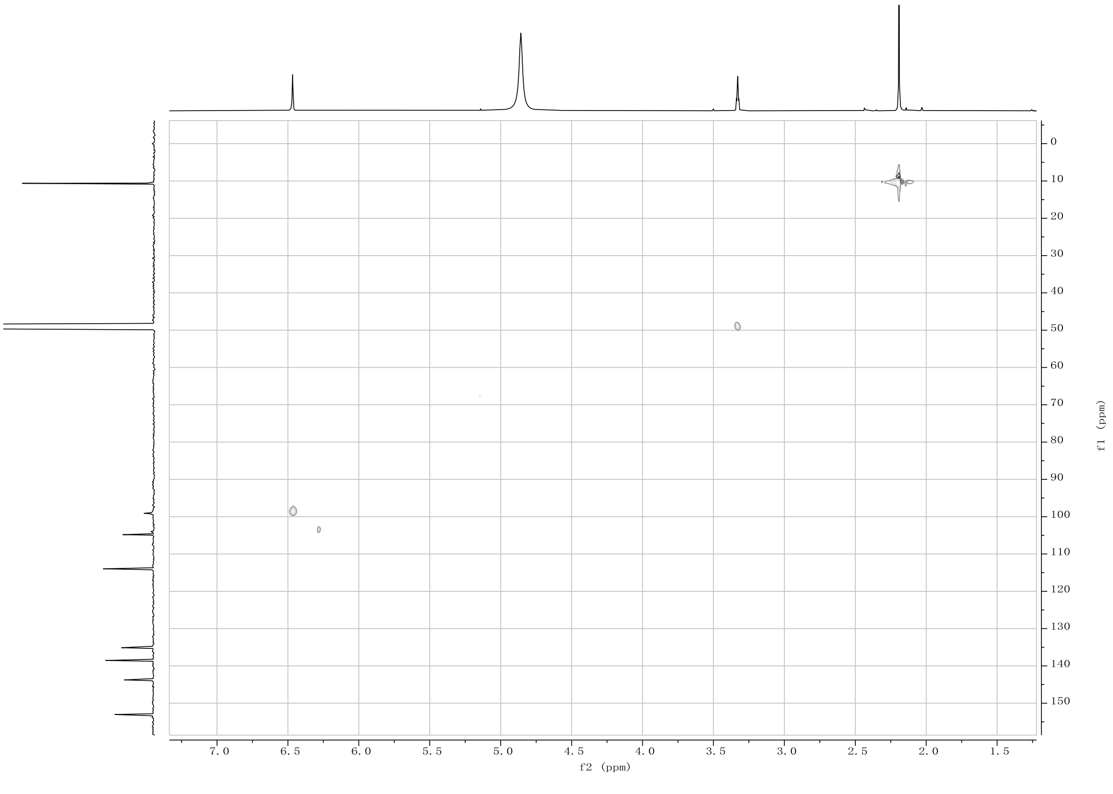


**Fig.S16.** HSQC spectrum of compound **3** (MeOH-$d_4$)

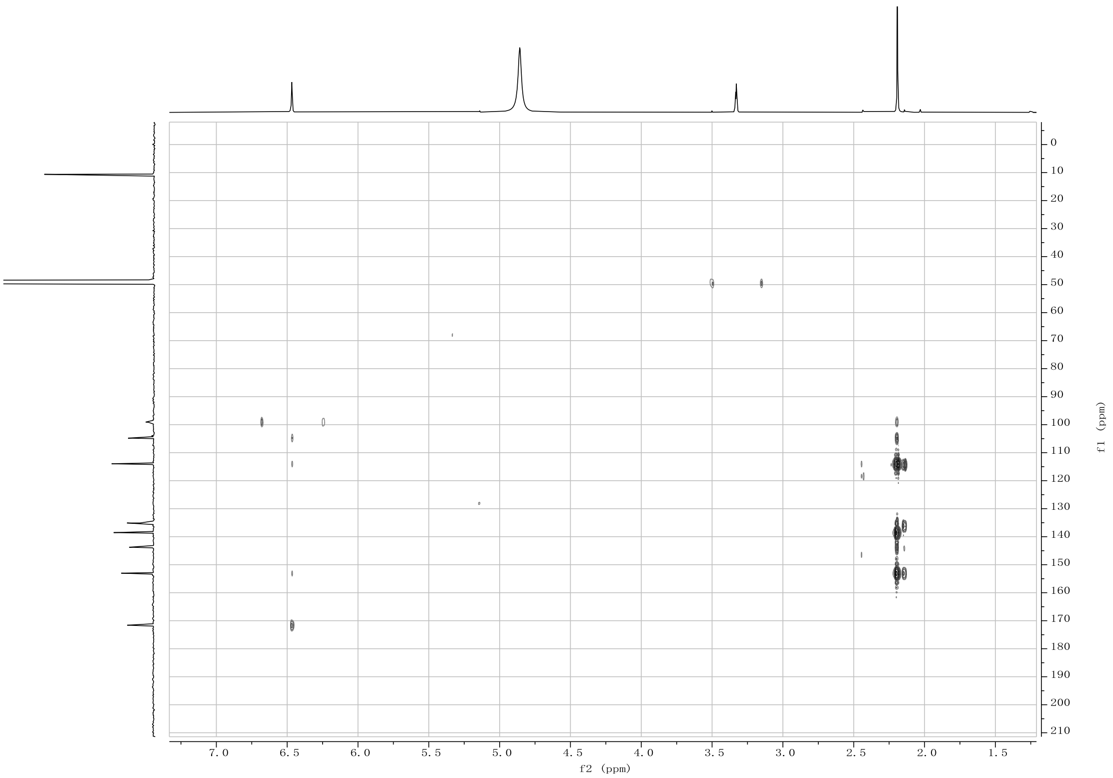


**Fig.S17.** HMBC spectrum of compound **3** (MeOH-$d_4$)
